# Longitudinal seroprevalence of Crimean-Congo hemorrhagic fever virus in Southern Uganda

**DOI:** 10.1080/22221751.2025.2465315

**Published:** 2025-02-13

**Authors:** Evan A. Mihalakakos, Victor Ssempijja, Ruy M. Ribeiro, Carmen Molina-Paris, Gerald Katushabe, Josephine Nalwadda, Jonah Omooja, Denis K. Byarugaba, Kyle Rosenke, Steven J. Reynolds, Mary K. Grabowski, Ronald M. Galiwango, Robert Ssekubugu, Heinz Feldmann, David W. Hawman

**Affiliations:** a Laboratory of Virology, Rocky Mountain Laboratories, NIAID/NIH, Hamilton, MT, USA; b Laboratory of Immunoregulation, NIAID/NIH, Bethesda, MD, USA; c Theoretical Biology and Biophysics Group, Theoretical Division, Los Alamos National Laboratory, Los Alamos, NM, USA; d Laboratory of Virology, NIAID/NIH International Centers for Excellence in Research, UVRI, Entebbe, Uganda; e College of Veterinary Medicine, Makerere University, Kampala, Uganda; f Department of Pathology, Johns Hopkins School of Medicine, Baltimore, MD, USA; g Rakai Health Sciences Program, Kalisizo, Uganda

**Keywords:** CCHF, seroprevalence, Uganda, antibodies, Africa

## Abstract

Crimean-Congo hemorrhagic fever (CCHF) is a tick-borne disease endemic to many regions of Africa, the Middle East, Southeast Asia and the Balkans. Caused by the CCHF virus (CCHFV), CCHF has been a recognized cause of illness in Uganda since the 1950s and recently, more intensive surveillance suggests CCHFV is widely endemic within the country. Most surveillance has been focused on the Ugandan cattle corridor due to the risk of CCHFV exposure associated with livestock practices. Here we evaluated the seroprevalence of CCHFV in several Southern Ugandan communities outside the cattle corridor combined with longitudinal sample sets to measure the immune response to CCHFV for up to a decade. Interestingly, across three community types, agrarian, trading and fishing, we detected CCHFV seroprevalence in all three but found the highest seroprevalence in fishing communities. We also measured consistent CCHFV-specific antibody responses for up to a decade. Our findings support the conclusion that CCHFV is widely endemic in Uganda and highlight that additional communities may be at risk for CCHFV exposure.

## Introduction

**Background –** Crimean–Congo hemorrhagic fever virus (CCHFV) is a negative-sense enveloped RNA virus belonging to the *Bunyavirales* order [1,2]. CCHFV was first identified in the Congo during the 1960s and soon after in the Crimean Peninsula [3,4]. Today, CCHF is the most widespread tickborne viral hemorrhagic fever in the world; endemic in Africa, the Middle East, Southeast Asia, and the Balkans [5]. The distribution of CCHFV follows the geographic distribution of its primary vector and reservoir host, the *Hyalomma* tick [6]. Ticks acquire CCHFV through vertical transmission from parent to offspring or horizontal transmission during co-feeding [7]. Wild mammals and livestock act as amplifying hosts, playing a crucial role in the persistence of CCHFV in the environment. CCHFV is transmitted to humans primarily through tick bites or contact with blood or tissues of viremic animals. High-risk exposure exists for individuals with outdoor activities as well as those working with livestock, including herders, butchers, and veterinarians [8–10]. Healthcare and intrafamily transmission have been reported but are rare [11–13]. While CCHFV can infect multiple wild and domestic animal species, only humans develop symptomatic disease [1]. The clinical presentation of Crimean – Congo hemorrhagic fever (CCHF) in humans varies widely, ranging from asymptomatic and mild infections to severe hemorrhaging and lethal disease [14]. Due to the non-specific symptoms and milder cases of CCHF, cases of CCHF may be significantly underreported in endemic regions. For recognized cases, case fatality varies by geographic region but can exceed 30% [15].

Between 1958 and 1978, Uganda reported its earliest cases of CCHFV followed by a significant absence of known cases until 2013 [16]. The resurgence of cases since then could be attributed to various factors, including biological vectors, human risk factors, and/or Uganda’s advancing surveillance and diagnostic capacity [17,18]. In Uganda, the seroprevalence of CCHFV IgG antibodies has been reported to be as high as 91.8% in livestock and 27.6% in humans [19]. Spatial prediction modelling estimates a nationwide livestock seropositivity rate of 30% [20]. Most CCHFV serosurveys have focused on the “cattle corridor”, a savannah grassland stretching from the North-Eastern to the South-Western parts of Uganda. The area is densely populated with herding and trading livestock, creating a potential network for the spread of CCHFV [21]. This could explain why the region holds the majority of historical CCHFV cases and why, from 2013 to 2019, animal handlers accounted for 65% of all confirmed cases [9]. Nevertheless, the epidemiology of CCHFV outside the cattle corridor is poorly understood, where tick dynamics, livestock practices, and climate may differ.

In this report we aimed to advance the understanding of CCHFV in Southern Uganda by assessing seroprevalence and identifying associated risk factors among agrarian, trade, and fishing communities largely outside the Ugandan cattle corridor. We performed a cross-sectional survey of human sera (n = 1,199) collected by Rakai Health Sciences Program’s (RHSP) Rakai Community Cohort Study (RCCS), and evaluated for CCHFV exposure by several laboratory and statistical methods. In addition, longitudinal samples spanning up to a decade from CCHFV-seropositive individuals were evaluated to measure the antibody response to CCHFV over time. Together our data suggests that CCHFV is circulating in Uganda outside the high-risk “cattle corridor” while also indicating that antibody responses to CCHFV are maintained for years, potentially for life, after infection. Cumulatively, our report adds to our understanding of the epidemiology of CCHFV in Uganda and suggests CCHFV is widely endemic in the country.

## Materials and methods

**Study Population**-The Rakai Community Cohort Study (RCCS), ongoing since 1994 by RHSP, is a longitudinal population-based cohort of ∼20,000 individuals aged 15–49 years in the Masaka region of Southern Uganda [22,23]. At ∼18-month intervals, RCCS holds a census of all residents, whether permanent or transient, in every household from ∼40 agrarian, trading, and fishing cohort communities. After the census, structured confidential interviews are conducted to consenting individuals aged 15–49 years followed by collection of a blood sample, GPS coordinates, and sociodemographic and health data.

**Study Design** – This CCHFV serological screening was a retrospective study nested within the RCCS round 19 cohort, which was conducted from June 2018–October 2020. To detect a CCHFV seroprevalence of 1% (± 1% with 95% confidence) required a minimum of 381 sera samples per the three community types (trading, agrarian, fishing) which was rounded off to sampling 400 participants per sub-population of interest totalling 1,200 participants. Selection followed a two-stage stratified-random sampling procedure. The first stage of sampling stratified the study communities into three strata based on the dominant economic activity of agrarian, trading or fishing. Twenty-one (21) communities classified as predominantly agrarian, 16 predominantly trading, and 4 predominantly fishing cohort communities. In the second stage, we randomly selected 400 HIV negative participants from the three strata. This resulted in a sampling ratio of 1:18, 1:14 and 1:6 in the agrarian, trading and fishing communities respectively. One individual randomly selected from the trading community cohort did not consent to further research with their sample and was removed from the study. After identifying CCHFV IgG positive individuals at round 19, longitudinal sera samples from the same individuals were tested from RCCS round 16 (July 2014-January 2015), round 17 (February 2015–September 2016), round 18 (October 2016–May 2018), and round 20 (February 2021-March 2023). In addition, any available round 19 samples from household contacts of positive individuals were tested.

**Testing for CCHFV serology:** A two stage-serial testing algorithm was applied as follows: First, sera samples were heat-inactivated at 56°C for 1 hour and then screened by the ID Screen® CCHF Double Antigen ELISA (Innovative Diagnostics) for CCHFV nucleoprotein IgG antibodies according to manufacturer’s instructions. This assay has documented high-specificity and sensitivity and is able to distinguish between CCHFV and closely related *Orthonairoviruses* [24]. Sera samples with S/P% (S/P% = (OD of the sample (OD_S_)/OD of the positive control (OD_PC_)) x 100) below or equal to 30% were classified as negative and S/P% over 30% as positive per kit instructions. In addition to kit provided controls, known CCHFV IgG positive human serum from The Public Health Agency of Sweden was used as a positive control. Blank wells and pooled Normal Human Serum (Innovative Research) were used as negative controls.

Samples positive by the ID Screen® ELISA were then screened by an in-house ELISA for secondary assay confirmation and calculating IgG endpoint titres. 96-well Nunc Maxisorp plates were coated with irradiated CCHFV whole-virus (strain Hoti) from tissue-culture supernatant [25] diluted at 1:1000 in PBS and incubated overnight at 4°C. Antigen was removed, plates blocked with 0.1% milk in PBS + Tween then incubated at room temperature for 30 minutes. Block was washed 3x with 250ul of PBS + Tween. 100ul of the sera diluted in 0.1% milk at 1:400 was added in duplicate to the plates followed by serial four-fold dilutions, 1:1600; 1:6,400; 1:25,600; 1:102,400; 1:409,600; 1:1,638,400. Blank wells, known CCHFV IgG positive human sera, and negative pooled human sera were added in duplicate to each plate for controls. Plates were incubated at room temperature for 1 hour then washed 3x with 250ul of PBS + Tween. 100ul of secondary human antibody biotin conjugate (Goat Anti-Human IgG-BIOT, Cat. No.: 2040-08, SouthernBiotech) diluted at 1:4000 in PBS + Tween was added to each well. Plates were incubated at room temperature for 1 hour and then washed 3x with 250ul of PBS + Tween. 100ul of Streptavidin-HRP conjugate (Streptavidin-HRP Cat. No.: 7105-05, SouthernBiotech) diluted to 1:4000 in PBS was added to each well. Plates incubated at room temperature for 30 minutes then washed 6x with 250ul of PBS + Tween. 100 μL/well of ABTS® 2-Component Microwell Peroxidase Substrate containing 2, 2'-azino-di (3-ethylbenzthiazoline-6-sulfonate, Material Number 5120-0032, SeraCare) at 1:1 dilution was added, and the plates incubated in the dark for 30 minutes. Optical density (OD) was read spectrophotometrically at 415 nm and averaged by the duplicate wells. The IgG titre endpoint cut-off value was determined using the negative human serum sample OD415 nm average and standard deviation among the 36 plates tested as defined by the following equation: cut-off = 36-plate mean OD415 nm + (3 × 36-plate standard deviation). The highest dilution with a signal above the determined cut-off absorbance value was assigned as a sample’s IgG endpoint titre. Samples were only considered positive if they were positive in both the commercial ID Screen® and in-house ELISA. There was strong agreement between the ID Screen® ELISA and our in-house ELISA. One sample positive by the ID Screen® ELISA was negative in our in-house assay (endpoint > 1:100), however, this sample was barely above the cutoff value in the ID Screen® assay. This sample was considered negative for all subsequent analyses.

**Neutralization assay.** Neutralization activity of sera against CCHFV strain UG3010 was performed as previously described [26]. As a positive control we used a known neutralizing monoclonal antibody 11E7 [27] that exhibited a neutralization titre of 1:1920 against CCHFV strain UG3010 (data not shown).

**Statistical analysis:** The seroprevalence of CCHFV in each community was determined independently for each stratum (community type) by the proportion of individuals testing CCHFV seropositive in the study sample. Population level CCHFV seroprevalence was determined by weighted proportions which were compared between strata using a weighted Pearson χ2 statistic obtained by applying a second-order Rao and Scott correction factor to the simple Pearson χ2 statistic. We also evaluated the determinant factors of CCHFV burden with a weighted generalized linear model with stratified weights computed as the ratio of the strata population size and the sample size and population correction factor that was computed as the inverse of the stratified survey weights. The linearized variance – covariance matrix was used to account for correlation. We also performed a secondary analysis to measure the interaction effect of community type and livestock ownership of cows, goats or pigs.

For study participants that tested CCHFV IgG seropositive at RCCS round 19, we conducted a longitudinal study to assess CCHFV IgG antibody half-life decay by retrospectively testing samples from RCCS survey rounds 16-20.

**Longitudinal IgG Titer Statistical analysis-** We quantified the rate of decline of the antibodies after study entry, assuming an exponential decay in titres over time. This assumption was consistent with visual inspection of the data. The model fitted was a simple exponential decay given by

$$\mathrm{Ab}=Ab_{0}e^{-\lambda_{1}t},$$

where *Ab* is the antibody titre (as a function of time), *Ab*_0_ is the titre from which the decay begins (at time zero), and $\lambda_{1}$ is the (positive) rate of decay. This model assumes that time 0 is the time at which the participant entered the study. It is possible that this occurs at different times post-infection for each individual. However, the stability of the levels and the consistency of decay rate across the majority of individuals indicates this is a reasonable assumption for time 0.

For the fits, we used a linear mixed effects modelling approach using the package lmerTest of the statistical computing software R. In this approach the antibody data from all the study participants was fitted simultaneously, on a log_10_ scale. We, thus, estimate population level parameters for *Ab*_0_ and $-\lambda_{1}$, assuming that each person is representative of the overall population of interest. We allowed for random effects both in the intercept and slope of the regression, but the best model included random effects only in the intercept.

**Mapping CCHFV prevalence and case counts-** Maps were generated on Datawrapper using RCCS round 19 community GPS coordinates and CCHFV serostatus from participants residing in the 41 communities from June 2018–October 2020.

**Ethics-** The Rakai Community Cohort Study (parent study) was approved by the Research and Ethics Committee of the Uganda Virus Research Institute (GC/127/19/03/709), the Uganda National Council for Science and Technology (HS-364), and the Johns Hopkins School of Medicine Institutional Review Board (IRB00204691). All participants signed written informed consent prior to enrolment.

**Data sharing-** De-identified Rakai Community Cohort Study data can be provided to interested parties subject to the completion of the Rakai Health Sciences Program data request form and the signing of a Data Transfer Agreement. Inquiries should be directed to datarequests@rhsp.org.

## Results

**Study Population Demographics-** The 1,199 study participants came from 21 agrarian communities (n = 400), 16 trading communities (n = 399), and 4 Lake Victoria fishing communities (n = 400) in Southern Uganda. Demographic composition of the study sample can be found in Table 1 while that of the weighted population distributions can be found in Supplementary Table 1. We observed no significant difference in the demographic distribution of the sample versus weighted population. Therefore, we reported the primary results based on the study sample while the weighted study results were mainly reported in the supplementary results.

**Table 1.** Individual demographic characteristics of RCCS round 19 participates by agrarian, trade, and fish landing site communities from June 2018–October 2020 (n = 1,199).

Variable	Agrarian community	Trading community	Fishing community	*p*-value
				
*Overall*	**400(100%)**	**399**(**100%)**	**400**(**100%)**	
				
*Gender*				
Female	202(51%)	233(58%)	172(43%)	**<0**.**001**
Male	198(50%)	166(42%)	228(57%)	
*Age (years)*				
15–24	163(41%)	174(44%)	112(28%)	**<0**.**001**
25–34	110(28%)	112(28%)	156(39%)	
>35	127(32%)	113(28%)	132(33%)	
*Education*				
None	10(3%)	7(2%)	21(5%)	**<0**.**001**
Primary	198(50%)	146(37%)	254(64%)	
Secondary/Tertiary	192(48%)	246(62%)	125(31%)	
*Occupation*				
Agriculture	168(42%)	113(28%)	28(7%)	**<0**.**001**
Housework/Unemployed	14(4%)	35(9%)	27(7%)	
Formal/Government	24(6%)	23(6%)	12(3%)	
Alcohol/Gambling/Sexwork	5(1%)	13(3%)	34(9%)	
Casual labour	30(8%)	35(9%)	15(4%)	
Small business	64(16%)	89(22%)	114(28%)	
Student	61(15%)	59(15%)	10(3%)	
Fishing	2(1%)	0(0%)	115(29%)	
Other	32(8%)	32(8%)	45(11%)	
*Own goats*				
No	262(66%)	296(74%)	368(92%)	**<0**.**001**
Yes	138(35%)	103(26%)	32(8%)	
*Own cows*				
No	342(86%)	335(84%)	369(92%)	**<0**.**001**
Yes	58(14%)	64(16%)	31(8%)	
*Own pigs*				
No	191(48%)	226(57%)	351(88%)	**<0**.**001**
Yes	209(52%)	173(43%)	49(12%)	

**Figure 1.**
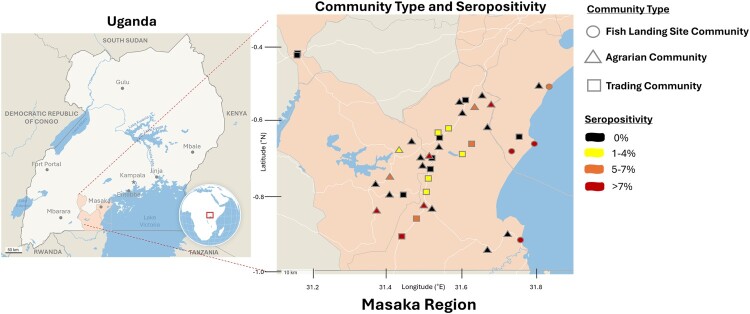
Seropositivity of CCHFV in Southern Uganda. Map of Uganda with highlighted Masaka region where RCCS study samples were collected. CCHFV seropositivity map by community type (circle = fish landing site, triangle = agrarian, square = trading; black = 0%, yellow = 1-4%, orange = 5–7%, red = >7%).

**Seroprevalence of CCHFV by community type-** Among the 1,199 samples, we found an average seropositivity of 7.75% (range: 5.4-11.6%) in fishing communities, 2.75% in agrarian (range: 0-12.5%), and 2.25% (range: 0-8.3%) in trading communities for an overall seroprevalence of 4.25% (51/1,199) (Table 2), while the weighted population seroprevalence was 3.3% (Supplementary Table 2). Community seropositivity and GPS coordinates are in Figure 1. All three community types had a significant difference when comparing the burden of CCHFV seroprevalence (*p*-value < 0.001). Agrarian versus trading communities had low and similar prevalence of CCHFV (*p*-value = 0.655). In combination, agrarian and trading communities had a significantly lower prevalence of CCHFV at 3% compared to fishing communities with a prevalence of 8% (*p*-value < 0.001). Since agrarian and trading communities were statistically significantly similar by serostatus and to improve the testing power, the communities were pooled for subsequent analysis by demographic variables. The distribution of demographic characteristics for pooled agrarian/trading communities versus fishing communities differed by all demographic characteristics including age, gender, education level, occupation, and differed by cow, goat or pig ownership (Supplemental Table 3). Notably, fishing communities had more males, were older, had lower educational status and primarily held occupations in fishing and small business.

**Table 2.** Community level burden of CCHFV.

	Community type			Overall
Exposure Variable	Agrarian	Trading	Fishing	
*CCHFV status*				
No	389(97%)	390(98%)	369(92%)	1148(96%)
Yes	11(3%)	9(2%)	31(8%)	51(4%)
*Chi-squared p-values*				
Agrarian vs Trading vs Fishing	<0.001			
Agrarian vs Trading	0.655		–	
Exposure variable	Agrarian or Trading		Fishing	
*CCHFV status*				
No	779(97%)		369(92%)	
Yes	20(3%)		31(8%)	
*Chi-squared p-values*				
Agrarian/Trading vs Fishing	<0.001			

**Factors Associated with CCHFV Status-** Using multivariate analysis, we assessed demographic factors associated with infection of CCHFV (Table 3) and found that fishing community residence and small business work were significantly associated with CCHFV serostatus. Residing in fishing communities was associated with a 2.7-fold higher risk of CCHFV when compared to residing in agrarian or trading communities (adjusted prevalence ratio = 2.66, 95% CI = 1.18-5.97). Small business work was associated with a 2.7-fold decrease in the risk of CCHFV when compared to agriculture work. Multivariate analysis of the stratified weighted population showed individuals >35 years old associated with a 3.06-fold higher risk of CCHFV when compared to individuals 15–24 years old (Supplemental Table 4). Given the recognized risk of CCHFV exposure from livestock, we assessed the interaction effect of cows, pigs or goats’ ownership and the community type in the risk of CCHFV infection (Table 4). We found that ownership of cows and pigs was associated with an increased burden of CCHFV in the fishing communities, while goat ownership was not associated with the burden of CCHFV in the agrarian or trading communities. Interestingly, not owning goats in fishing communities was associated with an increased risk of CCHFV. Weighted population results did not differ in significance (Supplemental Table 5).

**Table 3.** Factors associated with CCHFV status.

Variable	CCHFV status % (n/N)	Univariate		Multivariate	
		uPRs (95% CIs)	p-value	aPRs (95% CIs)	p-value
*Community type*	** **				
Agrarian or Trading	2.5%(20/799)	Ref		Ref	
Fishing	7.8%(31/400)	**3.10**(**1.79–5.36)**	**<0**.**001**	**2.65**(**1.18–5.97)**	**0**.**018**
*Gender*	** **				
Female	3.6%(22/607)	Ref		Ref	0.914
Male	4.9%(29/592)	1.35(0.79–2.33)	0.277	1.04(0.49–2.21)	
*Age (years)*	** **				
15–24	2.2%(10/449)	Ref		Ref	
25–34	4.0%(15/378)	1.78(0.81–3.92)	0.151	1.16(0.49–2.72)	0.736
>35	7.0%(26/372)	**3.14**(**1.53–6.42)**	**0**.**002**	1.95(0.85–4.47)	0.113
*Education*	** **				
None	5.3%(2/38)	Ref		Ref	
Primary	6.5%(39/598)	1.24(0.31–4.94)	0.761	1.69(0.43–6.67)	0.451
Secondary/Tertiary	1.8%(10/563)	1.24(0.31–4.94)	0.151	1.69(0.43–6.67)	0.656
*Occupation*	** **		.		.
Agriculture	5.5%(17/309)	Ref		Ref	
Housework/Unemployed	6.6%(5/76)	1.20(0.46–3.14)	0.717	1.14(0.38–3.43)	0.809
Formal/Government	3.4%(2/59)	0.62(0.15–2.60)	0.510	0.90(0.20–4.06)	0.890
Alcohol/Gambling/Sexwork	5.8%(3/52)	1.05(0.32–3.45)	0.938	0.69(0.15–3.15)	0.633
Casual labour	3.8%(3/80)	0.68(0.20–2.27)	0.532	0.76(0.21–2.69)	0.665
Small business	2.6%(7/267)	0.48(0.20–1.13)	0.093	**0.34**(**0.13–0.93)**	**0**.**036**
Student	0.8%(1/130)	0.14(0.02–1.04)	0.055	0.34(0.04–2.99)	0.332
Fishing	10.3%(12/117)	1.86(0.92–3.78)	0.085	0.71(0.28–1.81)	0.468
Other	0.9%(1/109)	0.17(0.02–1.24)	0.080	0.12(0.01–1.01)	0.051
*Own goats*	** **				
No	5.1%(47/926)	Ref		Ref	
Yes	1.5%(4/273)	0.91(0.40–2.10)	0.828	1.43(0.64–3.21)	0.383
*Own cows*	** **				
No	4.3%(45/1046)	Ref		Ref	
Yes	3.9%(6/153)	**0.29**(**0.10–0.79)**	**0**.**016**	0.40(0.14–1.19)	0.100
*Own pigs*	** **				
No	4.9%(38/768)	Ref		Ref	
Yes	3.0%(13/431)	0.61(0.33–1.13)	0.117	0.94(0.44–1.99)	0.871

uPR = univariate prevalence ratio; aPR = adjusted prevalence ratio.

**Table 4.** Interaction of livestock ownership and community type with CCHFV status.

Variable	CCHFV status % (n/N)	Univariate		Multivariate	
		uPRs (95% CIs)	p-value	aPRs (95% CIs)	p-value
*Goat ownership versus community type*					
No goats: agrarian/trading	3.0%(17/558)	Ref		Ref	
No goats: Fishing	**8.2%**(**30/368)**	**2.68**(**1.50–4.78)**	**<0**.**001**	**2.63**(**1.14–6.05)**	**0**.**023**
Goats: agrarian/trading	1.2%(3/241)	0.41(0.12–1.38)	0.15	0.38(0.11–1.36)	0.139
Goats: fishing	3.1%(1/32)	1.03(0.14–7.47)	0.98	1.21(0.17–8.59)	0.851
*Cow ownership versus community type*					
No cows: agrarian/trading	2.7%(18/677)	Ref		Ref	
No cows: Fishing	**7.3%**(**27/369)**	**2.75**(**1.54–4.93)**	**<0**.**001**	2.23(0.96–5.21)	0.063
Cows: agrarian/trading	1.6%(2/122)	0.62(0.14–2.63)	0.513	0.76(0.19–3.05)	0.699
Cows: fishing	**12.9%**(**4/31)**	**4.85**(**1.75–13.49)**	**0**.**002**	**5.11**(**1.74–14.97)**	**0**.**003**
*Pig ownership versus community type*					
No pigs: agrarian/trading	3.1%(13/417)	Ref		Ref	
No pigs: Fishing	**7.1%**(**25/351)**	**2.28**(**1.19–4.40)**	**0**.**013**	1.87(0.81–4.31)	0.144
Pigs: agrarian/trading	1.8%(7/382)	0.59(0.24–1.46)	0.252	0.54(0.21–1.41)	0.209
Pigs: fishing	**12.2%**(**6/49)**	**3.93**(**1.56–9.87)**	**0**.**004**	**3.28**(**1.28–8.39)**	**0**.**013**

uPR = univariate prevalence ratio; aPR = adjusted prevalence ratio adjusted for age, education, and occupation.

**Figure 2.**
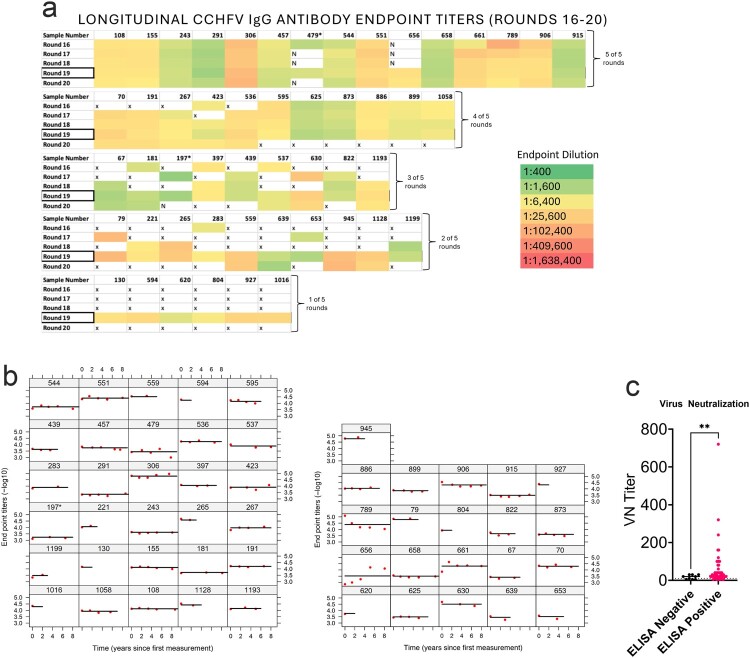
Antibody responses to CCHFV are lifelong. (a) Longitudinal CCHFV IgG antibody endpoint titres of the 51 positive individuals with sera collected between July 2014–March 2023, RCCS rounds 16–20 respectively. Each box represents 1 individual titled by their sample number and colour coded to their CCHFV-specific IgG endpoint titre. *Sample numbers 479 and 197 were weakly positive at round 19 cross-sectional testing, which may explain the low or negative titres longitudinally. (b) The fitted line is the antibody titre decay data of all individuals simultaneously using a mixed-effect regression approach. Available longitudinal sample sets of seropositive individuals by number of rounds are as follows- 1 round:6 individuals, 2 rounds:10 individuals, 3 rounds:9 individuals, 4 rounds:11 individuals, 5 rounds:15 individuals. (c) Sera was evaluated for neutralization against authentic CCHFV strain UG3010. VN titres are reported as the reciprocal of the last dilution to show no cytopathic effect. *P* value calculated with Welch’s t-test. ** *P* < 0.01.

**Longitudinal titers to CCHFV.** The RCCS performs repetitive rounds of sampling roughly every 18 months with low participation attrition. We therefore evaluated longitudinal samples from CCHFV positive individuals from round 16 (sampling conducted in 2014) to round 20 (2022). Not all CCHFV seropositive individuals participated in all 5 rounds, but we were able to evaluate 45 seropositive individuals over two or more rounds (Figure 2a). Six seropositive individuals participated only in our initial round 19 cross sectional analysis. Overall, IgG endpoint titres remained generally consistent through rounds 16–20 suggesting that antibodies against CCHFV persisted in the blood for nearly a decade, the longest timeframe tested (Figure 2a-b). However, one individual seroconverted between June 2017-June 2019 (Figure 2a, #656). Information on any febrile illness this individual may have had between round 18 (2017) and round 19 (2019) is not part of the standard RCCS questionnaire and therefore is unknown.

To calculate the durability of these antibody responses, we fitted the antibody titre decay data of all individuals simultaneously using a mixed-effect regression approach (Figure 2b). The model assumed exponential decay and provided a very good description of the longitudinal antibody titres in most individuals. The population estimate of antibody half-life was ∼58 years (Figure 2b), indicating negligible decay over the lifetime of the person. A small number of individuals seem to have a different longitudinal pattern. For example, participant numbers 656 and 661 show an increase of Ab over early time (Figure 2b), which could be consistent with recent exposure as participant 656 seroconverted between rounds 18 and 19 or re-exposure. Further, participant number 789 had a relatively fast decay of Ab titres (Figure 2b), which could also indicate a recent infection and titre plateau prior to the sample collected during round 16.

**Serum from seropositive individuals is poorly neutralizing.** To further evaluate the antibody response to CCHFV, sera from ELISA seropositive and 9 randomly chosen negative individuals from the round 19 cohort were evaluated for neutralization against authentic CCHFV strain UG3010. Although CCHFV strain UG3010 was isolated in the 1950s, CCHFV strain UG3010 is genetically similar to strains of CCHFV sequenced from Ugandan CCHF cases identified in 2018–2019 [9]. Although seropositive individuals had significantly increased neutralizing titres compared to seronegative individuals (Figure 2c), median neutralization titres of seropositive individuals against infectious CCHFV were low (1:25) compared to a median neutralization titre of 1:20 in seronegative individuals (Figure 2c). The one individual who seroconverted between round 18 and round 19 (Figure 2a), had a neutralization titre of 1:100 suggesting that recent CCHFV infections may not result in long-term neutralizing antibody responses. As our initial screen utilized an NP-specific assay while neutralizing antibodies against CCHFV target the Gc protein [27], we cannot exclude the possibility that we excluded individuals with Gc dominant responses. However, in a recent report from Uganda, all individuals had reactivity to both NP and Gc [28] and the high-specificity and sensitivity of the initial screening assay when evaluated against a large panel of known positive CCHFV cases [24] argues against this explanation. Instead, our data suggest that exposure to CCHFV elicits durable antibodies against the nucleoprotein while neutralizing responses against the viral glycoproteins may be weak or wane over time.

**Figure 3.**
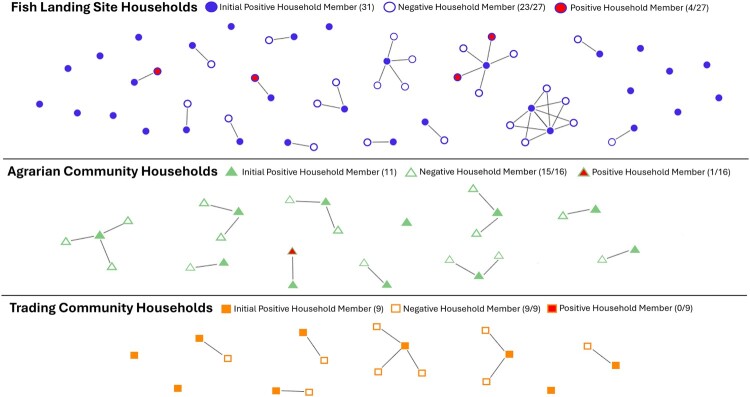
Household contact network stratified by fish landing site, agrarian, and trading community households. Of the 52 household contacts tested, 5 were seropositive for CCHFV IgG antibodies. 1 household had 2 index positive individuals. Line = household contact connection. Red shape = positive household contact. Filled shape = index positive household member from 1,199 sample set. Empty shape = negative household contact.

**Household Contacts-** The RCCS collects samples with household identification, and we were also able to evaluate samples of close household contacts of our index CCHFV seropositive individuals. Of the 51 index positive individuals, 31 index positive individuals had at least one household contact to test for a total of 52 household contacts. Of the 52 household contacts available, five were seropositive for CCHFV, while two index individuals were from the same household. In total, five of the 31 households with at least one available contact had ≥2 positive members. Notably, three individuals from one household were seropositive for CCHFV. However, due to the small number of positive household contacts it is unclear if household contacts of seropositive individuals are at increased risk of CCHFV exposure through shared environmental risk factors or human-to-human transmission. A visual of the household contact network stratified by community type can be found in Figure 3.

## Discussion

Our investigation of CCHFV seropositivity among people living in Southern Uganda support the wide endemicity of CCHFV in Uganda. We identified a total average seropositivity of 4.25% (51/1,199; range: 0-12.5%) among trading (9/399; range: 0-8.3%), agrarian (11/400; range: 0-12.5%), and fishing (31/400; range: 5.4-11.6%) communities within the Masaka region of Southern Uganda. Our results align with other human serology studies in Uganda that have reported seropositivity rates of 6.0%, 2.2%, 10·3% [29–31]. Such rates suggest CCHFV cases are highly underreported, despite the progress in Uganda’s surveillance capabilities. Various factors contributing to this include persistent misdiagnosis and limited access to healthcare [32,33]. CCHF symptoms can manifest similarly to malaria, other viral hemorrhagic fevers, and other common infectious diseases in Uganda leading to misdiagnosis. For example, during the 2022 Ebola Virus (EBOV) outbreak, four suspected EBOV cases were later confirmed to be PCR-positive for CCHFV. Over the five-month EBOV outbreak, there were a total of 13 confirmed CCHFV cases, including 7 deaths for a case fatality rate of 53.8% [34]. Also in 2022, an individual within the Masaka region was initially treated for resistant malaria until their hemorrhagic symptoms worsened triggering the UVRI surveillance system and later confirmed CCHFV positive [35]. Together, these cases plus our retrospective serological results suggest that subclinical CCHFV infections may routinely occur in Uganda while also suggesting that symptomatic CCHF cases may be misdiagnosed or go unrecognized.

Surprisingly, in our study, we identified fishing communities as having the highest seropositivity. To our knowledge, this is the first study investigating CCHFV burden in this community type. Additionally, cow and pig ownership within the fishing communities showed significantly increased risk of infection compared to agrarian and trade communities. With livestock ownership as a common practice among the three community types and Uganda’s lowland lake basins supporting high tick densities [36], further investigation is required to determine whether this risk difference is attributed to tick exposure along Lake Victoria, abattoir methods or both. In the absence of approved vaccines, public health education on risk factors associated with CCHFV exposure, implementing tick-control practices and personal protective equipment for high-risk occupations are the only effective measures to reduce CCHFV burden [1] . Our data suggest that even outside the Ugandan cattle corridor, Ugandans are frequently exposed to livestock and associated practices that may place them at risk for acquiring CCHFV and warrant inclusion of these communities in CCHFV public health education campaigns. Moreover, housing in fishing communities often include crowded, makeshift shelters comprised of timber and grass whose collection in the forests and bushes possibly increases exposure to ticks and also proximity to livestock [37]. Extensive longitudinal research conducted by RHSP has also revealed this population is unique in their demographic makeup and health behaviours compared to trading and agrarian communities including more males, older, mobile lifestyle, lower educational status, and hotspots for HIV incidence [22,38]. Our primary cohort did not include HIV-positive individuals, as we initially screened HIV-negative individuals. However, of the 11 HIV-positive household contacts 2 were also CCHFV seropositive. Further studies evaluating whether HIV status impacts CCHFV prevalence are warranted. Our results emphasize the need to explore the ecological, environmental, and cultural risks of CCHFV within the fishing communities so that targeted interventions can be implemented. The RCCS cohort enabled us to evaluate CCHFV seropositivity in close household contacts of positive individuals in our initial screen, but future studies will be needed to capture larger numbers of index cases and household contacts to more fully understand intra-household risks. Transmission of CCHFV in intrafamily settings have been reported but is rare [11–13,39] and most households in our study had only one seropositive individual.

Additionally, due to many individuals participating in the RCCS round over round, we were able to evaluate the antibody response to CCHFV over time. Our data suggest that antibody responses to CCHFV are long-lived, potentially for the life of the individual. Our results align with previous studies showing IgG presence in survivor sera up to 8 years following infection [40–43]. A recent report from Uganda evaluating responses to CCHFV up to 10 years after recognized symptomatic infection similarly showed persistent CCHFV-specific antibody responses that did not statistically wane over time [28]. Our population estimate of the antibody half-life was ∼58 years, indicating negligible decay over the lifetime of a survivor while suggesting the durability of immune response may be similar in subclinical and clinically recognized infections. No human CCHFV reinfection has been documented, although subclinical or asymptomatic reinfections likely would go unrecognized [44]. Whether antibodies or additional immune responses are responsible for this protection of survivors is unknown. Long-lived T-cell responses to CCHFV have also been reported and cellular immunity may also contribute to protection against reinfection [45–48]. Further, for all but one individual, our longitudinal sample set did not capture seroconversion suggesting that most CCHFV infections in our study population were not recent. Thus, how many years prior to sampling the individuals were exposed to CCHFV is unknown.

Interestingly, despite persistent anti-NP antibody responses, these sera exhibited poor neutralizing activity against a strain of CCHFV similar to recent isolates of CCHFV in Uganda. Cohen et al. in evaluation of serum collected from individuals with defined infections demonstrated neutralization activity at early timepoints post-infection that waned over time and also appeared strain-specific [28]. These findings may explain the poor neutralization activity measured in our cohort as it is unclear when post-infection our samples were collected but, in some individuals, could be greater than a decade. These cumulative data support the hypothesis that while NP-specific antibodies persist for decades, neutralizing antibody responses directed against the Gc may decline following CCHFV infection. However, it is unclear what strain of CCHFV our study participants were exposed to and genetic variability in the M segment of Ugandan CCHFV isolates [9] may have reduced neutralizing activity.

Our study has several important limitations. First, our sera sample set comprised of HIV-negative individuals and thus while two of the eleven subsequent household contacts were both CCHFV seropositive and HIV positive, significance could not be determined. As HIV presents a significant public health burden in Uganda, further research is needed to determine if HIV infection or covariate risk factors with HIV status correlates with CCHFV infection. Furthermore, although we evaluated serum samples across up to a 10-year timeframe, the year of seroconversion for most individuals could not be determined. Therefore, it is unknown how long after infection our samples were collected. It is also unclear if our CCHFV seropositive individuals developed symptomatic CCHF and many of the symptoms of CCHF may be attributed to other endemic diseases in Uganda [1]. The standard RCCS questionnaire would not capture data on febrile illnesses that could be attributed to CCHFV infection.

## Conclusion

In this study, we explored the prevalence and risk factors associated with CCHFV infections in the Masaka region of Southern Uganda. Our data add to the increasing evidence that CCHFV is widely endemic in Uganda and suggest that CCHFV is circulating outside the suspected high-risk “cattle corridor.” As risk-factors for CCHFV such as tick-exposure and livestock ownership commonly occur throughout Uganda, our findings are important to inform public health strategies within Uganda and in other regions where CCHFV may be circulating. In addition, our data demonstrate the value of the RHSP RCCS as a resource to investigate both the contemporary and historical prevalence and incidence of pathogens circulating within Uganda.

## Supplementary Material

Supplemental Material
