# Supplementary material for: Longitudinal seroprevalence of Crimean-Congo hemorrhagic fever virus in Southern Uganda
Source: Emerg Microbes Infect. 2025 Feb 13;14(1):2465315. doi: 10.1080/22221751.2025.2465315 (PMC11878160; doi:10.1080/22221751.2025.2465315)
Supplement: Supplemental Material [file TEMI_A_2465315_SM2702.docx]

| **Supplementary table 1: Demographic characteristics of participants in a stratified weighted study population** | | | | | |
| --- | --- | --- | --- | --- | --- |
| **Variable** | | **Agrarian community** | **Trading community** | **Fishing community** | **p-value** |
|  |  |  |  |  |  |
| **Overall** | | **7391(48%)** | **5687(37%)** | **2441(16%)** |  |
|  |  |  |  |  |  |
| **Gender** | |  |  |  |  |
|  | Female | 3732(51%) | 3321(58%) | 1050(43%) | 0.001 |
|  | Male | 3659(50%) | 2366(42%) | 1391(57%) |  |
| **Age (years)** | |  |  |  |  |
|  | 15-24 | 3012(41%) | 2480(44%) | 684(28%) | 0.002 |
|  | 25-34 | 2033(28%) | 1596(28%) | 952(39%) |  |
|  | >35 | 2347(32%) | 1611(28%) | 806(33%) |  |
| **Education** | |  |  |  |  |
|  | None | 185(3%) | 100(2%) | 128(5%) | <0.001 |
|  | Primary | 3659(50%) | 2081(37%) | 1550(64%) |  |
|  | Secondary/Tertiary | 3548(48%) | 3506(62%) | 763(31%) |  |
| **Occupation** | |  |  |  |  |
|  | Agriculture | 3104(42%) | 1611(28%) | 171(7%) | <0.001 |
|  | Housework/unemployed | 259(4%) | 499(9%) | 165(7%) |  |
|  | Formal/government | 444(6%) | 328(6%) | 73(3%) |  |
|  | Alcohol trade/Gambling/Sexwork | 92(1%) | 185(3%) | 208(9%) |  |
|  | Casual labour | 554(8%) | 499(9%) | 92(4%) |  |
|  | Small business | 1183(16%) | 1269(22%) | 696(29%) |  |
|  | student | 1127(15%) | 841(15%) | 61(3%) |  |
|  | Fishing | 37(1%) | 0(0%) | 702(29%) |  |
|  | Other | 591(8%) | 456(8%) | 275(11%) |  |
| **Own goats** | |  |  |  |  |
|  | No | 4841(66%) | 4219(74%) | 2246(92%) | <0.001 |
|  | Yes | 2550(35%) | 1468(26%) | 195(8%) |  |
| **Own cows** | |  |  |  |  |
|  | No | 6319(86%) | 4775(84%) | 2252(92%) | 0.014 |
|  | Yes | 1072(15%) | 912(16%) | 189(8%) |  |
| **Own pigs** | |  |  |  |  |
|  | No | 3529(48%) | 3221(57%) | 2142(88%) | <0.001 |
|  | Yes | 3862(52%) | 2466(43%) | 299(12%) |  |

| **Supplementary Table 2: Community level burden of CCHFV in a stratified weighted study population** | | | | | |
| --- | --- | --- | --- | --- | --- |
|  |  | **Community type** | | | **Overall** |
| **Exposure Variable** | | **Agrarian** | **Trading** | **Fishing** |  |
| **CCHFV status** | |  |  |  |  |
|  | No | 7188(97.3%) | 5559(97.7%) | 2252(92.3%) | 14999(97%) |
|  | Yes | 203(2.8%) | 128(2.3%) | 189(7.8%) | 520(3%) |
| **Chi-squared p-values** | |  | | |  |
|  | Agrarian vs trading vs Fishing | <0.001 | | |  |
|  | Agrarian vs trading | 1.000 | | - |  |
|  |  |  |  |  |  |
| **Exposure variable** | | **Agrarian or Trading** | | **Fishing** |  |
| **CCHFV status** | |  | |  |  |
|  | No | 12746(97.5%) | | 14998(96.6%) |  |
|  | Yes | 332(2.5%) | | 521(3.4%) |  |
| **Chi-squared p-values** | |  | |  |  |
|  | Agrarian/trading vs Fishing | 0.005 | | |  |

| **Supplementary Table 3: Individual demographic characteristics of RCCS round 19 participates pooled by agrarian/trade versus fish landing site communities from June 2018–October 2020 (n=1,199)** | | | | |
| --- | --- | --- | --- | --- |
| **Variable** | | **Agrarian / Trading community** | **Fishing community** | **p-value** |
|  |  |  |  |  |
| **Overall** | | **799(67%)** | **400(33%)** |  |
|  |  |  |  |  |
| **Gender** | |  |  |  |
|  | Female | 435(54%) | 172(43%) | <0.001 |
|  | Male | 364(46%) | 228(57%) |  |
| **Age (years)** | |  |  |  |
|  | 15-24 | 337(42%) | 112(28%) | <0.001 |
|  | 25-34 | 222(28%) | 156(39%) |  |
|  | >35 | 240(30%) | 132(33%) |  |
| **Education** | |  |  |  |
|  | None | 17(2%) | 21(5%) | <0.001 |
|  | Primary | 344(43%) | 254(64%) |  |
|  | Secondary/Tertiary | 438(55%) | 125(31%) |  |
| **Occupation** | |  |  |  |
|  | Agriculture | 281(35%) | 28(7%) | <0.001 |
|  | Housework/unemployed | 49(6%) | 27(7%) |  |
|  | Formal/government | 47(6%) | 12(3%) |  |
|  | Alcohol trade/Gambling/Sexwork | 18(2%) | 34(9%) |  |
|  | Casual labour | 65(8%) | 15(4%) |  |
|  | Small business | 153(19%) | 114(28%) |  |
|  | student | 120(15%) | 10(3%) |  |
|  | Fishing | 2(0%) | 115(29%) |  |
|  | Other | 64(8%) | 45(11%) |  |
| **Own goats** | |  |  |  |
|  | No | 558(70%) | 368(92%) | <0.001 |
|  | Yes | 241(30%) | 32(8%) |  |
| **Own cows** | |  |  |  |
|  | No | 677(85%) | 369(92%) | <0.001 |
|  | Yes | 122(15%) | 31(8%) |  |
| **Own pigs** | |  |  |  |
|  | No | 417(52%) | 351(88%) | <0.001 |
|  | Yes | 382(48%) | 49(12%) |  |

| **Supplementary Table 4: Factors associated with CCHFV status in a stratified weighted study population** | | | | | | | |  |
| --- | --- | --- | --- | --- | --- | --- | --- | --- |
| *Variable* | | *CCHFV status % (n/N)* | *Univariate* |  | *multivariate* | |  | |
|  |  |  | *uPRs (95% CIs)* | *p-value* | *aPRs (95% CIs)* | | *p-value* | |
|  |  |  |  |  |  | |  | |
| **Community type** | |  |  |  |  | |  | |
|  | Agrarian or Trading | 2.5%(332/13078) | Ref |  | Ref | |  | |
|  | Fishing | 7.7%(189/2441) | 3.06(2.14-4.37) | 0.005 | 2.24(0.75-6.63) | | 0.086 | |
| **Gender** | |  |  |  |  | |  | |
|  | Female | 2.8%(229/8103) | Ref |  | Ref | |  | |
|  | Male | 3.9%(292/7416) | 1.39(0.79-2.45) | 0.126 | 1.32(0.39-4.41) | | 0.429 | |
| **Age (years)** | |  |  |  |  | |  | |
|  | 15-24 | 1.3%(81/6175) | Ref |  | Ref | |  | |
|  | 25-34 | 3.4%(157/4581) | 2.60(0.24-28.47) | 0.228 | 1.89(0.03-104.12) | | 0.567 | |
|  | >35 | 5.9%(282/4763) | 4.48(0.46-44.12) | 0.106 | 3.08(0.10-97.06) | | 0.295 | |
| **Education** | |  |  |  |  | |  | |
|  | None | 3.0%(12/412) | Ref |  | Ref | |  | |
|  | Primary | 5.4%(394/7289) | 1.83(0.06-57.50) | 0.53 | 2.68(0.06-123.24) | | 0.384 | |
|  | Secondary/Tertiary | 1.5%(114/7816) | 1.83(0.06-57.50) | 0.503 | 2.68(0.06-123.24) | | 0.938 | |
| **Occupation** | |  |  | . |  | | . | |
|  | Agriculture | 4.8%(235/4885) | Ref |  | Ref | |  | |
|  | Housework/unemployed | 3.3%(30/922) | 0.69(0.01-78.42) | 0.765 | 0.94(0.08-10.98) | | 0.924 | |
|  | Formal/government | 2.4%(20/844) | 0.50(0.02-16.61) | 0.485 | 0.81(0.01-86.63) | | 0.865 | |
|  | Alcohol/Gambling/Sexwork | 6.3%(30/484) | 1.31(0.08-21.12) | 0.714 | 1.14(0.03-50.88) | | 0.894 | |
|  | Casual labour | 2.3%(26/1144) | 0.48(0.02-10.64) | 0.415 | 0.54(0.03-10.25) | | 0.461 | |
|  | Small business | 2.1%(67/3147) | 0.44(0.03-5.89) | 0.309 | 0.36(0.02-8.26) | | 0.297 | |
|  | student | 0.9%(18/2029) | 0.19(0.01-3.84) | 0.14 | 0.65(0.01-82.72) | | 0.737 | |
|  | Fishing | 9.9%(73/739) | 2.06(1.17-3.62) | 0.031 | 0.63(0.32-1.23) | | 0.097 | |
|  | Other | 1.4%(18/1322) | 0.29(0.01-10.81) | 0.279 | 0.25(0.00-24.60) | | 0.323 | |
| **Own goats** | |  |  |  |  | |  | |
|  | No | 4.1%(463/11305) | Ref |  | Ref | |  | |
|  | Yes | 1.4%(57/4213) | 0.33(0.13-0.84) | 0.036 | 0.42(0.13-1.36) | | 0.086 | |
| **Own cows** | |  |  |  |  | |  | |
|  | No | 3.4%(459/13346) | Ref |  | Ref | |  | |
|  | Yes | 2.8%(61/2173) | 0.82(0.09-7.66) | 0.74 | 1.19(0.11-12.92) | | 0.78 | |
| **Own pigs** | |  |  |  |  | |  | |
|  | No | 4.1%(363/8892) | Ref |  | Ref | |  | |
|  | Yes | 2.4%(158/6627) | 0.58(0.28-1.22) | 0.089 | 0.75(0.18-3.09) | | 0.471 | |
| uPR=univariate prevalence ratio; aPR=adjusted prevalence ratio | | | | | |  | |  |

| **Supplementary Table 5: Interaction of animal ownership and community type with CCHFV status in a stratified weighted study population** | | | | | | |
| --- | --- | --- | --- | --- | --- | --- |
| *Variable* | | *CCHFV status % (n/N)* | *Univariate* |  | *multivariate* |  |
|  |  |  | *uPRs (95% CIs)* | *p-value* | *aPRs (95% CIs)* | *p-value* |
|  |  |  |  |  |  |  |
| **Goat ownership versus community type** | |  |  |  |  |  |
|  | No goats : agrarian/trading | 3.1%(280/9060) | Ref |  | Ref |  |
|  | No goats : Fishing | **8.1%(183/2246)** | **2.63(1.70-4.09)** | **0.011** | **2.22(0.76-6.44)** | **0.085** |
|  | Goats : agrarian/trading | 1.3%(51/4018) | 0.41(0.33-0.51) | 0.003 | 0.41(0.12-1.43) | 0.092 |
|  | Goats : fishing | 3.1%(6/195) | 1.01(0.65-1.57) | 0.931 | 1.12(0.49-2.55) | 0.606 |
| **Cow ownership versus community type** | |  |  |  |  |  |
|  | No cows : agrarian/trading | 2.7%(295/11095) | Ref |  | Ref |  |
|  | No cows : Fishing | **7.3%(165/2252)** | **2.76(2.65-2.86)** | **<0.001** | 1.94(0.72-5.24) | 0.103 |
|  | Cows : agrarian/trading | 1.9%(37/1984) | 0.70(0.02-22.42) | 0.703 | 0.87(0.03-25.09) | 0.876 |
|  | Cows : fishing | **12.9%(24/189)** | **4.86(4.68-5.05)** | **<0.001** | **4.46(1.43-13.92)** | **0.030** |
| **Pig ownership versus community type** | |  |  |  |  |  |
|  | No pigs : agrarian/trading | 3.1%(211/6751) | Ref |  | Ref |  |
|  | No pigs : Fishing | **7.1%(153/2142)** | **2.28(2.22-2.34)** | **<0.001** | 1.68(0.77-3.67) | 0.105 |
|  | Pigs : agrarian/trading | 1.9%(121/6328) | 0.61(0.20-1.88) | 0.200 | 0.57(0.20-1.59) | 0.142 |
|  | Pigs : fishing | **12.3%(37/299)** | **3.92(3.82-4.03)** | **<0.001** | **3.21(2.21-4.65)** | **0.005** |
| uPR=univariate prevalence ratio; aPR=adjusted prevalence ratio adjusted for age, education, and occupation | | | | | |  |
